# Environmental enrichment in middle age rats improves spatial and object memory discrimination deficits

**DOI:** 10.3389/fnbeh.2024.1478656

**Published:** 2024-10-17

**Authors:** Magdalena Miranda, Maria Carla Navas, Maria Belen Zanoni Saad, Dinka Piromalli Girado, Noelia Weisstaub, Pedro Bekinschtein

**Affiliations:** Laboratory of Memory Research and Molecular Cognition, Institute for Cognitive and Translational Neuroscience, Universidad Favaloro, Instituto de Neurología Cognitiva and CONICET, Buenos Aires, Argentina

**Keywords:** spatial memory, middle-aged, environmental enrichment, object memory, recognition, interference

## Abstract

Changes in memory performance are one of the main symptoms of normal aging. The storage of similar experiences as different memories (ie. behavioral pattern separation), becomes less efficient as aging progresses. Studies have focused on hippocampus dependent spatial memories and their role in the aging related deficits in behavioral pattern separation (BPS) by targeting high similarity interference conditions. However, parahippocampal cortices such as the perirhinal cortex are also particularly vulnerable to aging. Middle age is thought to be the stage where mild mnemonic deficits begin to emerge. Therefore, a better understanding of the timing of the spatial and object domain memory impairment could shed light over how plasticity changes in the parahipocampal-hippocampal system affects mnemonic function in early aging. In the present work, we compared the performance of young and middle-aged rats in both spatial (spontaneous location recognition) and non-spatial (spontaneous object recognition) behavioral pattern separation tasks to understand the comparative progression of these deficits from early stages of aging. Moreover, we explored the impact of environmental enrichment (EE) as an intervention with important translational value. Although a bulk of studies have examined the contribution of EE for preventing age related memory decline in diverse cognitive domains, there is limited knowledge of how this intervention could specifically impact on BPS function in middle-aged animals. Here we evaluate the effects of EE as modulator of BPS, and its ability to revert the deficits caused by normal aging at early stages. We reveal a domain-dependent impairment in behavioral pattern separation in middle-aged rats, with spatial memories affected independently of the similarity of the experiences and object memories only affected when the stimuli are similar, an effect that could be linked to the higher interference seen in this group. Moreover, we found that EE significantly enhanced behavioral performance in middle-aged rats in the spatial and object domain, and this improvement is specific of the high similarity load condition. In conclusion, these results suggest that memory is differentially affected by aging in the object and spatial domains, but that BPS function is responsive to an EE intervention in a multidomain manner.

## Introduction

Memory impairment is part of the aging process both in animals (Barnes et al., 1980; Barnes, 1988; Rapp and Amaral, 1992; Gage et al., 1988; Rapp and Heindel, 1994; Gallagher and Colombo, 1995; Lester et al., 2017) and humans (Rapp and Heindel, 1994; Lester et al., 2017; Craik, 1990; Gabrieli, 1996; Alexander et al., 2012). Aging has been associated with a disproportionate decrease in performance in particular cognitive domains, such as episodic and spatial memory (Lester et al., 2017; Barnes, 1979; Bohbot et al., 2012; Techentin et al., 2014; Isingrini and Taconnat, 2008; Spencer and Raz, 1995; Moffat, 2009; Bach et al., 1999; Ces et al., 2018; Creer et al., 2010; Rapp et al., 1997; Lacreuse et al., 2014; Moffat et al., 2006; Oler and Markus, 1998; Gallagher and Pelleymounter, 1988; Dunnett et al., 1988; Markowska et al., 1989; Cansino, 2009; O'Callaghan et al., 2009). The deficits found go from a reduced ability to maintain contextual details of an experience (Cansino, 2009; McDonough et al., 2014) to an increase in the susceptibility to interference (Talamini and Gorree, 2012) and a tendency to treat novel stimuli as familiar (ie. false recognition memory) (Yeung et al., 2014; Pidgeon and Morcom, 2014; Burke et al., 2010). All these deficits combine during aging, leading to retention impairments that start to emerge in middle-aged animals (Bizon et al., 2009; Driscoll et al., 2006; Foster et al., 2003; Kumar et al., 2012).

Recently, aging has begun to be considered a nonlinear process, with middle age signaled as the key point of acceleration in this progression (Dohm-Hansen et al., 2024). Human research suggests that one of the earliest behavioral expressions of this decline could be the reduced ability to discriminate similar stimuli in order to avoid interference, also referred as behavioral pattern separation (BPS) (Leal and Yassa, 2015). In fact, performance of memory discrimination tasks in humans was shown to be more sensitive to cognitive decline than standardized neuropsychological screening tools like the delayed recall RAVLT test (Stark et al., 2013). In rodents, the dentate gyrus (DG) region of the HP, is crucial for discrimination of overlapping spatial and non-spatial memory, having a key function in avoiding memory interference (Gilbert et al., 2001; Bekinschtein et al., 2013; Miranda et al., 2018; Miranda et al., 2017; Sahay et al., 2011).

Clear signs of memory decline are highly documented on rodents at a late aging stage (>20 months) (Barnes et al., 1980; Creer et al., 2010; Gallagher and Pelleymounter, 1988; Burke et al., 2012; Gage et al., 1984; Gallagher and Burwell, 1989; Holmes et al., 2010; Rapp et al., 1987; Johnson et al., 2017). However, it is currently believed that is actually during middle age that cognitive decline, and in particular spatial memory deficits, begin to emerge (Bizon et al., 2009; Begega et al., 2012; Frick et al., 1995). In contrast with this, there is a higher level of discrepancies in the impairments reported for non-spatial tasks, with reports of impairment in middle age (Bouet et al., 2011) that contrast with others showing no impairment in object recognition tasks until very late stages (>30 months) (Siette et al., 2013; Teglas et al., 2019). This could be the result of a mild level of mnemonic compromise in middle-aged animals (10 to 16 months old) (Calhoun et al., 1998; Das and Magnusson, 2008; Magnusson, 2001; Magnusson, 1998; Means and Kennard, 1991; Gros and Wang, 2018), that could only be evidenced in particular conditions, such as the high interference conditions present in BPS tasks.

A bulk of studies have suggested the use of a simple behavioral treatment, environmental enrichment (EE) (a multicomponent approach that provides sensory, motor, social and cognitive stimulation), could be effective in alleviating age-related memory dysfunction (Winocur, 1998; Harati et al., 2011; Sampedro-Piquero et al., 2013; Harburger et al., 2007; Harburger et al., 2007; Frick and Fernandez, 2003; Pham et al., 1999; Kempermann et al., 1998; Bennett et al., 2006; Lores-Arnaiz et al., 2006; Mirochnic et al., 2009; Mora et al., 2007; Soffie et al., 1999). In particular, EE has been shown to improve spatial cognitive decline (Kumar et al., 2012; Sampedro-Piquero et al., 2013; Harburger et al., 2007; Frick and Fernandez, 2003; Kempermann et al., 1998; Bennett et al., 2006; Speisman et al., 2013). On the other hand, EE has also proven to improve non-spatial object recognition learning abilities in aged animals (Gresack et al., 2007; Leal-Galicia et al., 2008; Cortese et al., 2018). Moreover, the emergence of studies that indicate that this beneficial effect of enrichment can be initiated at any point in the lifespan increases the therapeutic and translational utility of this approach (Sampedro-Piquero et al., 2013; Harburger et al., 2007; Bennett et al., 2006; Frick and Benoit, 2010; Rosenzweig and Bennett, 1996), with clinical trials showing benefits of enriching activities starting at old age (Ballesteros et al., 2015; Train the Brain Consortium et al., 2017; Cintoli et al., 2021; Ngandu et al., 2015). However, studies have shown that the magnitude of the effect may depend on the time of exposure being higher for middle-aged than aged animals (Kobayashi et al., 2002). In this sense, EE in middle-aged animals emerges as model of cognitive enrichment strategies in humans that, with the right timing, could help mitigate age-related cognitive impairments. Considering that BPS is one of the first functions to be affected by aging, understanding the differential contribution of EE to this function is crucial for the translational value of EE. However, little is known of how EE could differentially impact BPS in different memory domains.

In this work, we used middle-aged population to evaluate their cross-domain memory discrimination abilities under low and high interference memory conditions. For this purpose, we used variants of an incidental memory tasks (ie. object recognition/location tasks) where we quantitatively varied the load of similarity between the to-be-remembered objects in order to reveal subtle differences in memory discrimination performance both in the spatial and non-spatial domain. Here we describe a domain-specific impairment in BPS in middle-aged rats, with spatial memories affected independently of the similarity of the experiences and object memories affected as a function of the similarity load of the experience. Moreover, we studied the ability of EE as a therapeutic strategy to improve memory discrimination abilities in middle-aged rats and discovered that EE has beneficial effects on BPS performance both in the spatial and the object domain.

## Methods

### Subjects

The subjects were 81 Long-Evans male and female rats from our breeding colony. Young animals were 2–3 months old while middle-aged animals were 11–13 months old.

The rats were housed on a reversed 12-h light/12-h dark cycle (lights on 1900–0700), in groups of two to four. All behavioral testing was conducted during the dark phase of the cycle. Rats were food deprived to no less than 90% of their free feeding weight to increase spontaneous exploration. Water remained available *ad libitum* throughout the study. All experimentation was conducted in accordance with the National Animal Care and Use Committee (CICUAL).

#### Environmental enrichment

Animals were housed in subsequently different environments, as follows: (i) the control condition (SE), correspond to the time when animals were housed in standard laboratory cages (33.5*45*21.5 cm) with two animals per cage; (ii) the environmental enrichment condition (EE), corresponds to the condition where 4–6 animals were housed at a time in a big cage with several stages (33.5*45*64.5 cm) and many bottles of water and food hoppers, tubes, and ramps that were repositioned twice a week and changed weekly, as previously described (Dorfman et al., 2013). Rats were caged in these conditions for 4 weeks (Frick and Fernandez, 2003; van Praag et al., 2000; Nithianantharajah and Hannan, 2006). As part of the EE experiment, animals were tested immediately before entering the cage (SE condition) and after 4 weeks in the cage (EE condition). All animals were tested for the s- and d-SOR, as well as the s- and d-SLR on each of these two opportunities, using different and novel objects on each of these sessions. The EE experiment began when animals were 11–12 months old. For the duration of the behavioral testing, animals remained in their housing conditions (ie. EE or SE), as depicted in Supplementary Figure S1A.

The “cognitive training” exposure protocol (CT) consisted of 5 trials of repeated exposure to similar objects (ie, objects that shared one feature AB and BC). Before the CT protocol, there was a 3-day 5 min habituation to the triangular environment for the SOR. After the habituation, animals were tested for the s-SOR task. The CT sessions started following the test of the s-SOR. The CT sessions were separated by 24 h, and lasted 5 min. Animals were placed in the triangular context and were exposed to a couple of objects that shared one feature. The objects were designed by joining two small junk object elements. On each exposure session, the objects were formed by different features that were never repeated during CT protocol of during training or test sessions of the SOR. Immediately after the CT exposure, animals were tested for the s-SOR.

#### Apparatus

For the spontaneous object recognition task (SOR), the triangular open field made of white foam board was used. Each wall was 60 cm long by 60 cm high. The circular open field (90 cm diameter, 45 cm high) used for the spontaneous location recognition task (SLR) was made of black plastic. Both open fields were situated in the middle of a dimly lit room. The walls of the triangular open field were higher to minimize the visual access to the distal cues in the room. The circular open field was surrounded by six spatial cues. The open field floor was always covered with wood shavings. A video camera was positioned over the arena, and sample and choice phases were recorded for later analysis. The objects for the SOR task were made of two different smaller objects, except for the extra-similar condition, in which they were made by three smaller objects [for examples of the objects used, see (Miranda et al., 2018; Miranda et al., 2017)]. Composite objects were made by attaching together two or three of the smaller items in the conditions described in the Results section. We always used different junk objects for our within-subject design (Miranda et al., 2018; Miranda et al., 2017). Junk object features offer different textures and curvy shapes that are not present in LEGO-based objects. For the SLR, the objects used were either soda cans or beer bottles from which the label had been removed. All objects were fixed to the floor of the open field with Blu-tack and cleaned with a 50% ethanol solution between sample and choice trials. For the SOR task, all three composite objects were aligned close to one of the walls of the arena, and positions within this line were pseudorandomly assigned.

#### Behavioral procedures

**Figure 1:**
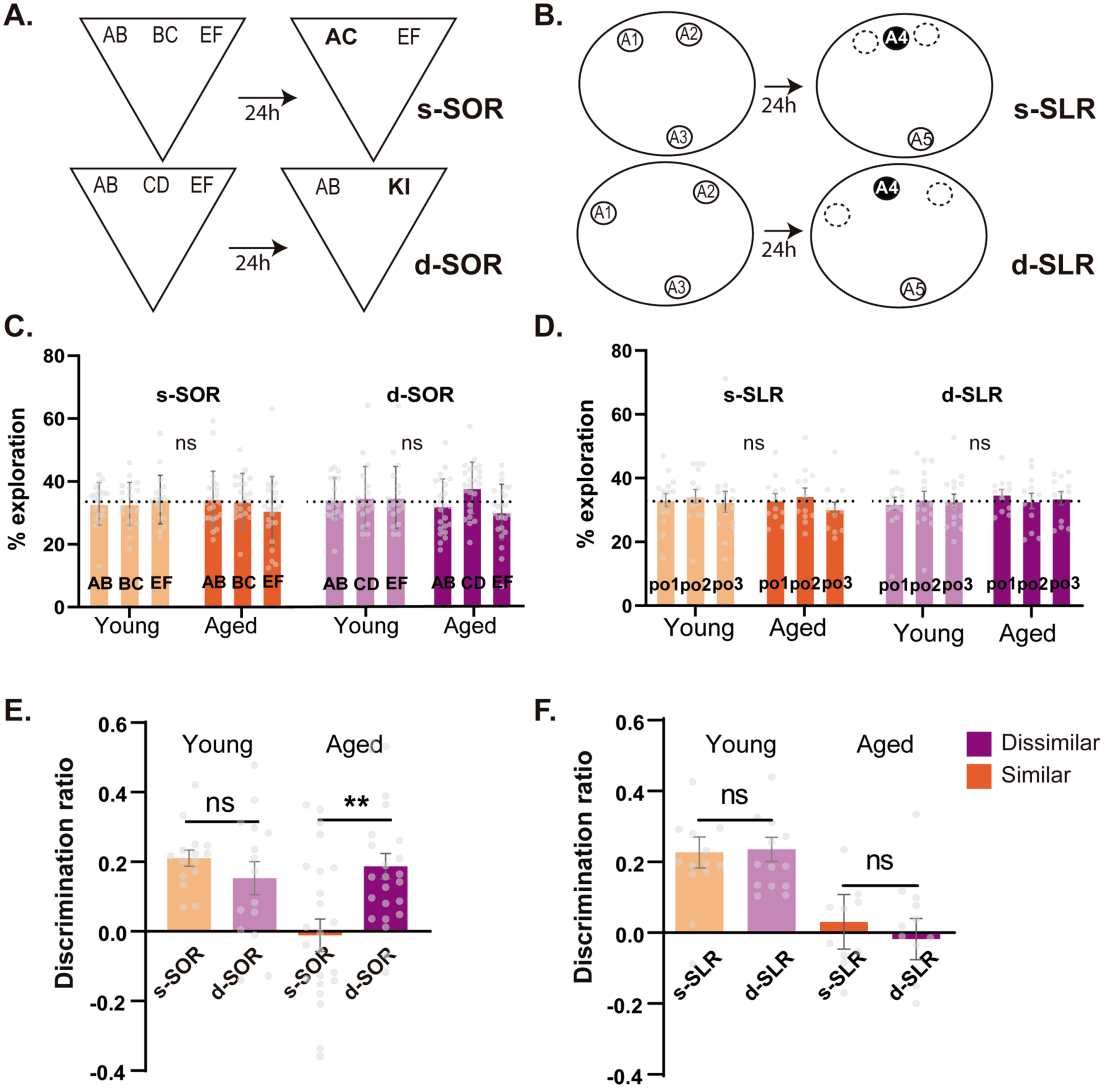
Early age-related impairment in object and spatial memory discrimination in high and low interference conditions. **(A)** Schematic illustration of the dissimilar (Down) and similar (Up) configurations of the spontaneous object recognition (SOR) task. Bold characters represent novel objects during choice session. **(B)** Schematic illustration of the two configurations of the SLR task, the dissimilar version (Down) and the similar version (Up), with black circles representing the novel position during the choice session. **(C)** Percentage of time animals spent exploring each object in the high similarity (s-SOR, orange) and low similarity (d-SOR, violet) conditions of the SOR task both in young (left) and middle-aged (right) animals. **(D)** Percentage of time animals spent exploring each object in the high similarity (s-SLR, orange) and low similarity (d-SLR, violet) conditions of the SLR task both in young (left) and middle-aged (right) animals. *n* = 19–24. **(E)** Performance of young and middle-aged rats in the s-SOR and d-SOR task. **(F)** Performance of young and middle-aged rats in the s-SLR and d-SLR task. *n* = 12–14. Data expressed as the mea*n* ± SEM; *p < 0.05, ** *p* < 0.01, # represents *p* < 0.05 against 0. Light colors represent the values for young animals and darks colors those of aged animals.

Before every experiment, all rats were handled for 2 days and habituated to each empty context during either 5 min over 3 days (SOR) or 10 min over 5 days (SLR). The SOR and SLR task were performed as previously reported (Miranda et al., 2018; Miranda et al., 2017; Miranda et al., 2021). Briefly, for the SOR task, after habituation, the rats were exposed during a 5-min duration sample phase to three objects made of either two or three features depending on the condition. For the similar condition, two of the objects shared one feature (AB and BC) and the third object was made of two other different features (EF). For the dissimilar condition, all three objects were made of different features (AB, CD, and EF). The choice phase lasted 3 min and was conducted 24 h after the finalization of the sample phase. In this case, the animals were exposed to two objects, one novel and one familiar, that varied in composition according to the condition evaluated. For the similar condition, the novel object was made of the two nonshared features of the objects presented in the sample phase (AC), and the familiar object was a copy of the third object (EF). For the dissimilar condition, the novel object was made of two novel features (GH), and the familiar object was a copy of one of the objects presented during the sample phase (AB, CD, or EF) (see schemas in Figure 1A). We always used different objects and features for the different trials. This means that letters do not indicate the use of shared object or features across trials but only within trials (ie. they are a representation of the features that compose each object during each trial). Different features (A, B, C, etc) were used to reproduce the same task conditions in the consecutive trials of the within subject design. The rationale behind the task was that if the rats were able to separate the two similar objects, their representations should be distinct and resistant to confusion; therefore, the rats should show preference for the novel object during the retrieval phase. However, if the representations of the two similar objects were not sufficiently separated, presentation of the new object would activate a familiar representation in memory and would thus not be distinguishable. The result would be that rats should behave as if the new object was familiar.

For the SLR task, after habituation, rats were exposed to three identical objects, A1, A2, and A3, during a sample phase that lasted for 10 min. For the similar SLR (s-SLR), objects A2 and A3 were placed 50° apart (20.5 cm between them) and object A3 at an equal distance from the other two. For the dissimilar SLR (d-SLR), objects A1, A2, and A3 were equidistant, 120° (49 cm between them) apart from each other. Twenty-four hours after the sample phase, rats were exposed to two new identical copies of the objects, A4 and A5, for 5 min. New identical copies were used to prevent the use of olfactory cues. During this choice phase, object A4 was placed in a familiar location (same position as in the sample phase) and object A5 was placed in a novel location. For the s-SLR task, the novel location was defined as a position exactly in between the ones in which objects A2 and A3 were located during the sample phase (see schemes in Figure 1B). For the d-SLR task, object A4 was placed in a familiar location and object A5 in a position equidistant to the previous locations of A2 and A3 (see schemas in Figure 1B).

For the experiments shown in Figure 1, either young or middle age animals were tested two times. In the case of the SLR task (Figures 1D–F), independent groups of young and middle-aged animals were tested for both the similar and dissimilar version of the SLR task. For the SOR task (Figures 1C–E), independent groups of young and middle-aged animals were tested for both the similar and dissimilar version of the SOR task.

**Figure 2:**
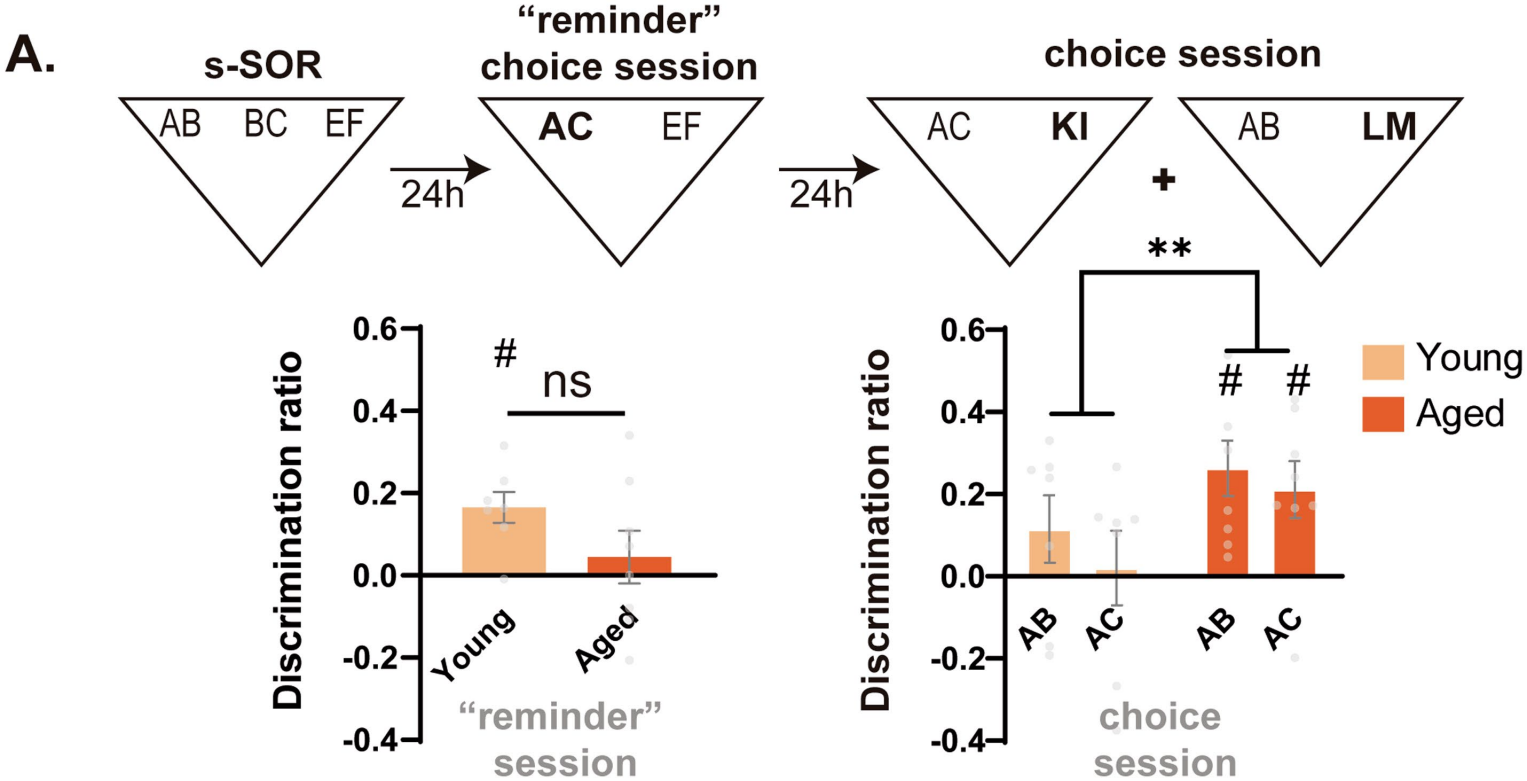
Early impairment in object memory discrimination is linked to memory interference in the aged group. **(A)** (Up) Timeline of the experiment. After animals went through a “reminder” choice session, memory for the AB and AC objects was tested against novel objects. (Down, left) Discrimination ratio of young and middle-aged animals during the “reminder” choice session. One sample t test against zero, Young *t* = 4.400 *p* = 0.005 Aged *t* = 0.693 *p* = 0.510. (Down, right) Discrimination ratio during the second test session 48 h after the original sample session. One sample *t* test AB young *t* = 1.401 *p* = 0.211, AC young *t* = 0.224 *p* = 0.830, AB aged *t* = 3.895, *p* = 0.006, AC aged *t* = 3.057 *p* = 0.018. Data expressed as the mea*n* ± SEM. Light colors represent the values for young animals and darks colors those of aged animals. *n* = 7–8, ** *p* < 0.01, # represents *p* < 0.05 against 0.

For the experiment of Figure 2, an independent set of animals was used. Animals were first trained in the similar version of the task. There was a reminder session 24 h after, where animals were presented with a familiar object (EF) and a similar novel object (AC), as during a normal choice test session. However, after this reminder, animals were retested twice, once for probing AB memory against a novel object and another for AC memory against a novel object. These two tests were given in a counterbalanced manner.

**Figure 3:**
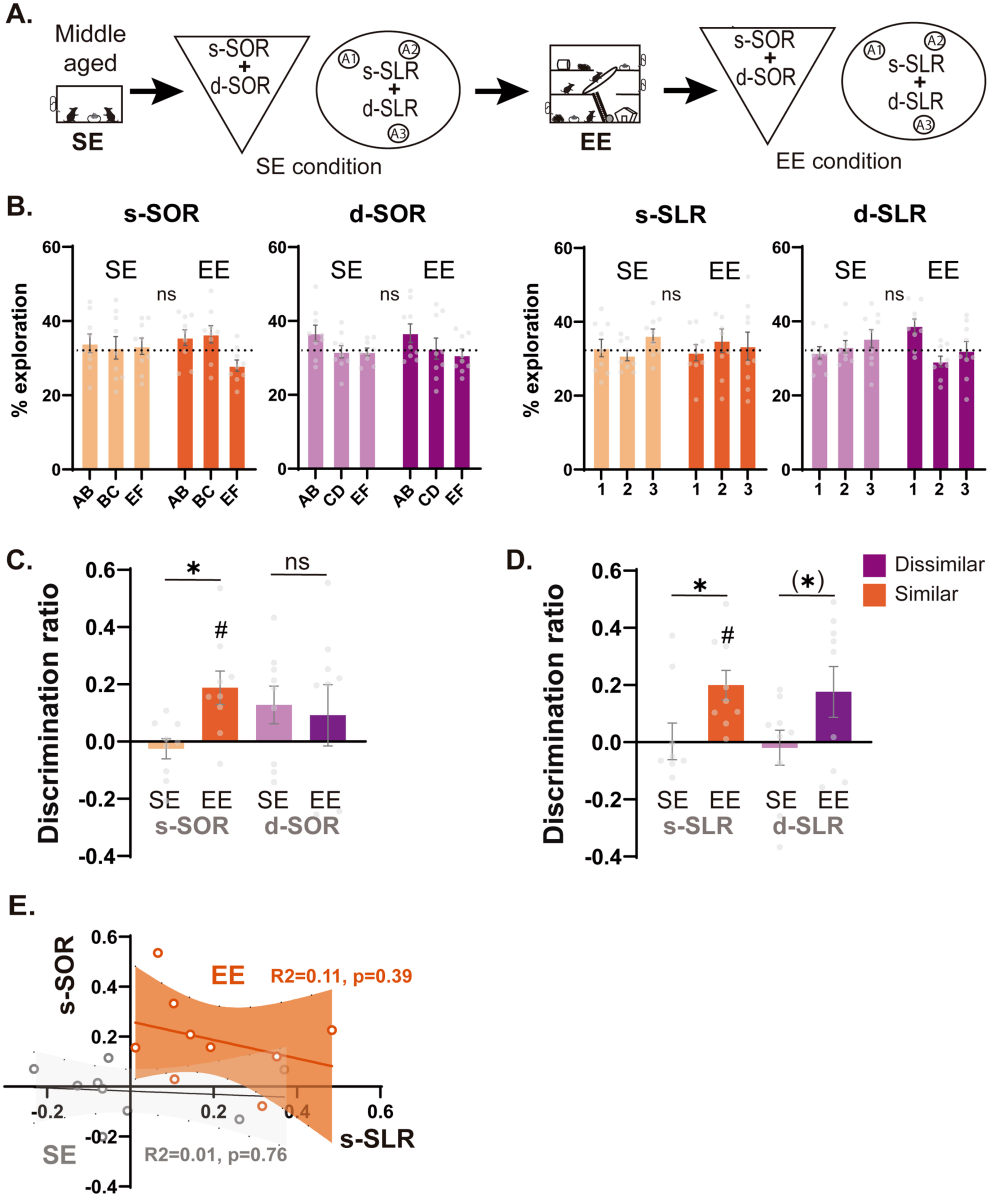
Effect of environmental enrichment over memory discrimination performance in low and high similarity conditions of the object and spatial domain. (A) Schematic representation of the task depicting the timeline of the experiment. One year old animals living in a standard environment were tested for the similar and dissimilar versions of both the SLR and SOR (SE condition, light) and after this they went through a 4-week continuous EE intervention. At the end of this month, they were re-tested under the same similar and dissimilar versions of the task (EE condition, dark). (B) Percentage of object exploration time in animals that went through the SOR task (Left) or SLR task (right) before (SE condition) or after (EE condition). (C) Performance of animals in the SE condition or EE condition for both the high similarity and low similarity versions of the SOR task. One sample t test against zero, s-SOR SE *t* = 0.726 *p* = 0.488, s-SOR EE *t* = 3.214 *p* = 0.012, d-SOR SE *t* = 0.194 *p* = 0.088, d-SOR EE *t* = 0.855 *p* = 0.418. (D) Performance of animals in the SE condition or EE condition for both the high similarity and low similarity versions of the SLR task. One sample t test against zero, s-SLR SE *t* = 0.046 *p* = 0.964, s-SLR EE *t* = 3.812 *p* = 0.005, d-SLR SE *t* = 0.312 *p* = 0.763, d-SLR EE *t* = 1.983 *p* = 0.082. (E) Linear regression between the discrimination ratios in the high similarity condition of the object task (s-SOR) and the high similarity condition of the spatial (s-SLR) task. Data expressed as the mea*n* ± SEM. Light colors represent the values for SE condition and darks colors those of EE condition. *n* = 9, *p < 0.05, # *p* < 0.05 against 0, (*) *p* < 0.07.

For the experiments shown in Figure 3, animals were tested 4 times (d-SOR, s-SOR, d-SLR, s-SLR) both before (SE condition) or after (EE condition) exposure to EE. In all the cases, the order of the tasks was counterbalanced.

Exploration was recorded and scored manually for both the sample and choice phases. For all experiments, exploration of a particular object was defined as the rat having its nose directed at the object at a distance of 2 cm or less, or touching the object with its nose. Rearing with the head oriented upward did not count as exploration. Climbing over or sitting on the objects was not included. Two people scored the videos; one was blind to the novel and familiar objects.

### Statistical analysis

For all the experiments, the results were expressed as a discrimination ratio that was calculated as the time exploring the novel object (SOR) or the object in the novel location (SLR) minus the time exploring the familiar object (SOR) or the object in the familiar location (SLR) divided by total exploration time [(t_novel_-t_familiar_)/t_total_]. One sample t tests were used to compare discrimination ratio from the similar and dissimilar conditions against zero. For the sample phase, the percentage of time exploring each object was compared between groups using a repeated measures two-way ANOVA, with age and object as the repeated measures. For the choice phase, discrimination ratios were compared within subject using a paired t test (s-SLR vs. d-SLR, s-SOR vs. d-SOR). In case of non-normal data, non-parametric analysis was used.

## Results

### Middle-aged animals are impaired on high similarity load object memory discrimination and both high and low similarity load spatial memory discrimination

To evaluate behavioral pattern separation in the object domain, a modified version of the spontaneous object recognition task was used (SOR), as in previous reports. The task was modified to evaluate the ability to discriminate objects with different degrees of similarity and is highly dependent on the PRC (Miranda et al., 2017; Miranda et al., 2021). In the object domain, this was achieved by controlling the load of object similarity using shared object elements, that were present in the similar version of the task (s-SOR, high load) but absent in the dissimilar version (d-SOR, low load) (see Methods) (Figure 1A).

During the choice session, we found that young animals performed correctly the object version of the task at both high and low load of similarity, as they showed positive discrimination indexes in both (One sample t test against 0; Young d-SOR t_(19)_ = 8.016, *p* < 0.0001; Young s-SOR t_(19)_ = 4.216, *p* = 0.0005) (Figure 1E). These results are in line with our previous work showing an ability in young rats to differentiate between similar object memories (Miranda et al., 2018; Miranda et al., 2017; Miranda et al., 2021). On the other side, middle-aged animals exhibited an impaired performance on the similar version of the SOR without an effect over the dissimilar version, as evidenced by its positive discrimination ratio [One sample *t* test against 0; s-SOR t_(24)_ = 0.248, *p* = 0.806; d-SOR t_(24)_ = 5.116 *p* < 0.0001] (Figure 1E). The performance on the s-SOR was significantly reduced when compared with the one of the d-SOR (Figure 1E). Further analysis revealed an interaction between age and condition, with significant differences between aged and young in the similar version of the task but not in the dissimilar [Two Way RM ANOVA, F_task (1,41)_ = 4.177 *p* = 0.047, F_age (1,41)_ = 5.707, *p* = 0.022, F_interaction (1,41)_ = 9.001, *p* = 0.005] (Figure 1E). Moreover, we found no differences in the total time aged animals spent exploring the objects on each version of the task during the sample sessions [Paired *t* test, t_(23)_ = 1.303 *p* = 0.202] or choice sessions [Paired *t* test, t_(22)_ = 0.733 *p* = 0.471] (Supplementary Table S1, S1.1). All in all, these results indicate a specific deficit in the discrimination of similar object memories in middle-aged rats.

Aging, as well as affecting memory functions, could also impair visual attention and perception (Jones et al., 1995; Muir et al., 1999; McGaughy and Sarter, 1995; Sarter and Turchi, 2002). The sample session phase of the SOR task has been designed as an additional high-demand perceptual/attentional task (ie. a variant of the perceptual oddity task), in which a reduction in the time exploring the similar objects (ie. AB and BC in s-SOR) can reveal a perceptual/attentional deficit that could prevent them to be identified as different objects (Bartko et al., 2007a; Bartko et al., 2007b). However, we found no significant differences between the amount of time the animals spent exploring each of the objects during sample phase in the s-SOR [Two way RM ANOVA, F_age(1,41)_ = 0.748 *p* = 0.392 F_obj(1.82, 74.41)_ = 0.168 *p* = 0.825, F_interaction(2,82)_ = 0.832 *p* = 0.439] nor the d-SOR [Two way RM ANOVA, F_age(1.72, 70.32)_ = 1.930 *p* = 0.158 F_obj(1,41)_ = 2.249 *p* = 0.141, F_interaction(2,82)_ = 1.728 *p* = 0.184] for aged compared to young animals during sample session (Figure 1C). This result suggests that middle-aged animals have no significant attentional/perceptual deficits in the object domain that could explain their impaired s-SOR performance.

In order to explore the effect of aging over memory discrimination in the spatial domain, we used an equivalent spatial version of the object task (the SLR task), in which the similarity relies on the distance between the positions of identical objects, leading to two conditions of high (small separation, s-SLR) and low (large separation d-SLR) spatial similarity (Bekinschtein et al., 2013; Miranda et al., 2018) (see Methods) (Figure 1B). During the choice session, we found that young animals could solve both the small and large versions of the task, as they show positive discrimination indexes in both [d-SLR t_(13)_ = 6.941, *p* < 0.0001; s-SLR t_(13)_ = 5.199, *p* = 0.0002] that did not differ significantly between d-SOR and s-SOR (Figure 1F). This confirms that young animals can distinguish between similar object positions, as previously reported (Bekinschtein et al., 2013; Miranda et al., 2018). In contrast, middle-aged animals showed a discrimination ratio not different from zero on both the similar and dissimilar versions of the SLR [d-SLR t_(11)_ = 0.310, *p* = 0.763; s-SLR t_(11)_ = 0.399, *p* = 0.697] (Figure 1F). Aged animals performed significantly worse than young animals independently of the similarity condition of the task [Two Way RM ANOVA, F_task(1,24)_ = 0.250 *p* = 0.622, F_age(1,24)_ = 12.16, *p* = 0.0019, F_interaction (1,24)_ = 0.515, *p* = 0.480] (Figure 1F). There were also no significant differences in the time spent exploring during sample session [Paired *t* test t_(11)_ = 1.722, *p* = 0.113] or choice session [Paired *t* test t_(11)_ = 0.559, *p* = 0.588] (Supplementary Table S1, S1.1). Moreover, there were also no differences in the amount of time spent exploring each object between the young and aged group on each task [RM Two Way ANOVA, s-SLR F_age(1,25)_ = 1.602 *p* = 0.217, F_obj(2,60)_ = 0.495 *p* = 0.613, F_interaction(2,50)_ = 0.100 *p* = 0.905, d-SLR F_age(1,25)_ = 2.122 *p* = 0.158, F_obj(2,50)_ = *p* = 0.011, F_interaction(2,50)_ = 0.214 *p* = 0.808] (Figure 1D). This result shows a highly impaired spatial memory discrimination performance (ie. both high and low similarity load), evident already at early stages of aging.

On whole, these results show that middle-aged rats have an affected spatial memory (independent of the load of similarity), while they exhibit a preserved object memory in which the deficits only manifest under a high load of object similarity.

Previous work has shown that, in conditions of high interference, memory impairment after medial temporal lobe damage can result not from the loss or inaccessibility of information but from novel information appearing as familiar (McTighe et al., 2010; Yeung et al., 2013). Taking this into account, we decided to explore whether the effect of aging on the object domain, which is dependent on the similarity, could be due to middle-aged animals falsely identifying the AC object as familiar. To explore this possibility, a new set of young and aged animals went through the s-SOR sample session and, 24 h after, they were subjected to a “reminder” choice session. The “reminder” choice session consisted of a short 3 min presentation to a novel yet similar AC object and a familiar EF object (as during the test in Figure 1E). Finally, 24 h after the “reminder” session, the memory of AC and AB was assessed against a novel object (Figure 2). The rationale of this experiment is that, if middle-aged animals identify the AC object as familiar, the test session could act as a practice of the AB memory and reinforce it when compared with young animals. In a similar manner, this false identification could lead to an apparently better memory of the AC object than young animals (for whom a 3 min choice session is normally not enough to create a long term memory (Stefanko et al., 2009)). When we compared the memory of AB and AC 48 h after the sample, we found that aged animals showed significantly higher memory of both AB and AC than young animals at 48 h [Two Way RM ANOVA F_age(1,13)_ = 8.028 *p* = 0.014, F_AB-AC(1,13)_ = 0.643, *p* = 0.437, F_interaction(1,13)_ = 0.055, *p* = 0.818] (Figure 2A). This suggests that the failure of middle-aged animals to perform the similar version of the task is not due to forgetting but to an inability to differentiate AC from the prior AB mnemonic representation.

### Environmental enrichment improves both spatial and object memory discrimination in a similarity dependent manner

Next, we decided to investigate the capacity of an environmental enrichment (EE) protocol to improve both object and spatial domain mnemonic functions. For this purpose, a group of middle-aged rats in a standard environment (SE) was tested for both the spatial and object versions of the task. After this, animals were placed in EE for 4 weeks, and following this time animals were re-evaluated with new object sets for the same two tasks (Figure 2A). This longitudinal approach was chosen because of its enhanced sensitivity and power when compared with cross-sectional studies (Caprioli et al., 1991; Dellu et al., 1997) that better accounts for the high individual variability present in aging animals (Frick et al., 1995; Fischer et al., 1992).

In the object SOR task, during the sample session, the EE group showed a reduced total object exploration time independently of the task condition [F_task(1,8)_ = 0.014 *p* = 0.910, F_EE(1,8)_ = 31.65 *p* = 0.0005, F_interaction(1,8)_ = 1.099 *p* = 0.253] (Supplementary Table S1, S3.1). However, animals did not differ in the percentage of time they spent exploring each object before and after the EE intervention [s-SOR F_obj(1,8)_ = 1.773 *p* = 0.202, F_EE(2,16)_ = 1.992 *p* = 0.196, F_interaction(2,16)_ = 1.082 *p* = 0.363; d-SOR F_EE(1,8)_ = 3.960 *p* = 0.082, F_obj(2,16)_ = 3.101 *p* = 0.072, F_interaction(2,16)_ = 0.051 *p* = 0.950] (Figure 3B). There was no change in total exploration time during = the choice sessions when compared before and after the EE [Two Way RM ANOVA F_task(1,8)_ = 0.063 *p* = 0.808, F_EE(1,8)_ = 3.340 *p* = 0.105, F_interaction(1,8)_ = 0.260 *p* = 0.624] (Supplementary Table S1, S3.2). During the choice session, we found that EE increased performance for the s-SOR version of the task, but not for the d-SOR version (Figure 3C) [Paired *t* test Similar t_(8)_ = 4.226 *p* = 0.003, Dissimilar t_(8)_ = 0.312 *p* = 0.763]. On the contrary, a cognitive exposure training, where animals were exposed during 5 sessions to objects that share one feature, did not lead to an improvement in memory performance on the object recognition task (Fig. S1B).

In the spatial SLR task, during the sample session, total object exploration was reduced after EE compared to before EE while no differences in total object exploration were evidenced between task conditions [Two Way RM ANOVA F_task(1,8)_ = 0.174 *p* = 0.687, F_EE(1,8)_ = 7.462 *p* = 0.026, F_interaction(1,8)_ = 0.097 *p* = 0.764] (Supplementary Table S1, S3.1). Despite this difference, animals did not differ in the percentage of time spent exploring each object before and after the EE [s-SLR F_EE(1,8)_ = 1.441 *p* = 0.264, F_obj(1.47,11.83)_ = 0.583 *p* = 0.524, F_interaction(1.5,12.8)_ = 0.462 *p* = 0.588; d-SLR F_EE(1,8)_ = 0.301 *p* = 0.598, F_obj(1.48,11.87)_ = 1.906 *p* = 0.194, F_interaction(1.41,11.28)_ = 2.694 *p* = 0.121] (Figure 3D). There was also a reduction in the exploration time after the EE intervention during the choice session [Two Way RM ANOVA F_task(1,8)_ = 4.84 *p* = 0.059, F_EE(1,8)_ = 12.57 *p* = 0.007, F_interaction(1,8)_ = 1.686 *p* = 0.230] (Supplementary Table S1, S3.2). During the choice phase, EE significantly increased discrimination ratios in the similar version of the task while only trended to a significant increase in the dissimilar version (Figure 3D, Paired t test Similar t_(8)_ = 3.018 *p* = 0.017, Dissimilar t_(8)_ = 2.152 *p* = 0.064). When we compared the performance of animals before and after the EE intervention, we found no correlation between s-SOR and s-SLR scores before or after the intervention (Before R2 = 0.013 *p* = 0.766, After R2 = 0.106 *p* = 0.392) (Figure 3E). All in all, we found EE intervention has the potential to increase memory function in a similarity specific manner, with differential effects over the spatial and the object memory domains.

## Discussion

In this work, we compared the middle age progression of low and high similarity load memory deficits in the object and spatial domains. Contrary to the similarity-dependent susceptibility in the object domain, we describe a more generalized impairment in spatial domain at this early stage. Additionally, we performed experiments that suggest that this similarity dependent impairment in object recognition could be due to middle-aged animals incorrectly identifying a novel object as familiar. Moreover, we used EE as a model of experience-dependent plasticity to show that 1 month EE exposure can rescue the deficit of animals on tasks of high interference both in the object and in the spatial domain.

A bulk of studies have shown that spatial memory is particularly susceptible to the effects of aging (Barnes, 1979; Moffat et al., 2006; Harburger et al., 2007; Bennett et al., 2006; Gallagher and Nicolle, 1993), and some authors attribute this impairment to a failure in binding contextual elements of an experience (Maguire et al., 1996; Rosenbaum et al., 2001). However, there are also reports of object recognition failure in aged rats (Bartolini et al., 1996; Wiig and Burwell, 1998; de Lima et al., 2005; Pieta Dias et al., 2007; Platano et al., 2008). In particular, aging was related to an inability to detect more subtle changes in object position or features, an ability often referred to as behavioral pattern separation (BPS) (Marr, 1971; Rolls and Kesner, 2006). In humans, studies have reported that older subjects have less efficient pattern separation on both spatial (Stark et al., 2010; Holden and Gilbert, 2012) and object domain tasks (Toner et al., 2009; Yassa et al., 2011; Trelle et al., 2017). Correspondingly, failure in discrimination of similar object positions (Ces et al., 2018; Creer et al., 2010; Gracian et al., 2013; Marrone et al., 2011) and objects with overlapping features (Johnson et al., 2017; Burke et al., 2011) has also been described in aged rodents. Here, we explored in further detail the temporality of these changes. We found a deficit in spatial memory in middle-aged animals that was independent of the similarity load of the task, suggesting that spatial memory function *per se* was already altered at this early stage and that this deficit is not specific to the BPS function. Certainly, it remains possible that earlier time points or lower interference loads in the task would reveal a BPS specific impairment.

The spatial pattern separation function is linked to the presence of adult neurogenesis in the dentate gyrus (DG) of the hippocampus (HP) and adult born neuronal integration as part of its circuitry (Sahay et al., 2011; Clelland et al., 2009; Nakashiba et al., 2012). Although both the DG (particularly neurogenesis in the DG) (Driscoll et al., 2006; Small et al., 2004; Patrylo and Williamson, 2007; Encinas et al., 2011; Ben Abdallah et al., 2010; Drapeau et al., 2003) and perforant path (Yassa et al., 2011; Barnes and McNaughton, 1980; Geinisman et al., 1992; Smith et al., 2000) are crucially affected by the aging process, we did not see this translate into a differential similarity-dependent deficit of aging since at the time point chosen the spatial memory function itself showed alterations that prevented to reveal this deficit. The presence of defective plasticity mechanisms in the middle-aged rat HP could explain the effect seen over spatial memory. In fact, rigidity in spatial representations could lead to an inability to differentiate between spatial locations (Barnes, 1994), and this inability could be more accentuated toward later time points.

Normal cognitive decline has been related to reduced synapse formation, expression of neuroprotectors like neurotrophins, altered neurogenesis and plasticity (Burke et al., 2010; Gallagher and Nicolle, 1993; Encinas et al., 2011; Ben Abdallah et al., 2010; Lu et al., 2005; Mattson and Arumugam, 2018). Although several interventions were studied to prevent this decline, EE has shown a particular interest for its translational value as a model of cognitive stimulation in humans. Even though EE has reported benefits over spatial memory performance in aged rodents (Harati et al., 2011; Bennett et al., 2006), how these benefits are dependent on the similarity load of the task is not clear. Moreover, prior work highlights the possibility that earlier interventions (ie. middle age) could be necessary in order to achieve a reproducible effect. Recent work has shown that the effect of EE over memory discrimination abilities in young adult rodents is associated with an EE-dependent increase in adult hippocampal neurogenesis (Frick and Fernandez, 2003; Pham et al., 1999; Paylor et al., 1992; Kempermann et al., 1997; van Praag et al., 1999; Auvergne et al., 2002; Cao et al., 2004; Bruel-Jungerman et al., 2005; Trinchero et al., 2017). The work also shows that these changes favor the sparsity of the network and a higher level of remapping in the presence of small environmental changes (Ventura et al., 2024). To add up to these findings, previous research indicated that the ability to encode small environmental changes is also influenced by the presence of the brain derived neurotrophic factor (BDNF) (Bekinschtein et al., 2014), a plasticity related molecule that has proven to be increased in the HP of EE housed animals and associated with newborn neuronal survival (Choi et al., 2009; Meng et al., 2015). We could speculate that EE, through an increase in neurogenesis and/or BDNF, could also reduce the rigidity of the spatial representations in middle-aged rodents. In line with this idea, EE can prevent age-related decline in precursor cell activity in the DG (Kempermann et al., 2002), and accelerate the neuronal development and integration of adult born granule cells in the DG in a BDNF-dependent manner (Trinchero et al., 2017). In this regard, previous work has shown that EE reverses the age-associated decrease in the expression of hippocampal tPA, a protease that controls the conversion from the precursor proBDNF to its mature form (Nagappan et al., 2009; Pang et al., 2004), that would ultimately change the balance between apoptotic and neurotrophic effects (ie. promoting neurogenesis) (Kempermann et al., 1997) and LTD/LTP (Lu et al., 2005; Nagappan et al., 2009; Pang et al., 2004; Figurov et al., 1996; Ghosh et al., 1994; Teng et al., 2005; Woo et al., 2005). Moreover, aged rats with preserved spatial reference memory showed higher number of new neurons in comparison to rats with spatial memory impairments (Driscoll et al., 2006; Drapeau et al., 2003). Considering the extensiveness of the molecular mechanisms of action of EE, from synaptic plasticity and cellular atrophy (Duffy et al., 2001; Eckert and Abraham, 2013; Park and Bischof, 2013) to dendritic growth (Leggio et al., 2005) and neurotransmitter systems (Segovia et al., 2006; Nichols et al., 2007; Segovia et al., 2008; Segovia et al., 2008), it is not surprising that its cognitive effects are widespread across domains. However, the fact that we do not see an impact over the dissimilar condition speaks of a mechanism that specifically impact the ability of the brain to avoid interference.

On the other side, in the object recognition domain, previous work has shown a specific deficit in a high similarity object condition in advanced aged (24–25 months) (Johnson et al., 2017; Burke et al., 2011). Here we extended these data by showing that this deficit could be present at earlier stages of aging (ie, middle-aged). Although the literature suggests there could be a decline in the ability to discriminate novel stimuli during aging (Burke et al., 2010; Bartolini et al., 1996; de Lima et al., 2005; Bastin and Van der Linden, 2003; Davidson and Glisky, 2002; Prull et al., 2006; Toth and Parks, 2006), likely due to a “false recognition” (Burke et al., 2010; Toner et al., 2009; Norman and Schacter, 1997; Jacoby et al., 2005), part of the memory field considers that recognition memory judgments are preserved and only recollection is affected by aging (Spencer and Raz, 1995; Driscoll et al., 2006; Bastin and Van der Linden, 2003; Davidson and Glisky, 2002; Daselaar et al., 2006; Pitarque et al., 2016; Koen and Yonelinas, 2016; Koen and Yonelinas, 2014). According to the “false recognition” theory, aging object recognition impairment is related to a difficulty in differentiating a novel object when it shares features with familiar ones leading to interpret it as a familiar object (ie. reduced exploration of the novel object). This false recognition has been associated with a higher susceptibility to interference occurring during the delay period (Burke et al., 2010; Trelle et al., 2017; Burke et al., 2011). In accordance with this theory, we described a pattern of memory practice effects that paradoxically increase object recognition memory only in middle-aged animals. This result evidences that the lack of discrimination of similar objects in middle-aged animals is not due to a retrieval deficit, as memory the next day is even higher for the middle-aged group compared to controls. More likely, this deficit is due to an incorrect identification of the AC object as familiar, what leads to a “practice-driven” increase in recognition of AB a day later.

This work has chosen a longitudinal approach for evaluating the role of EE. This choice could lead to a potential confounding effect of practice over the results presented. However, we do not believe this effect could explain our results since we have shown that the repeated presentation (or “practice”) of objects of similar identities over extended periods of time does not lead to an increase in the performance in aged animals. This result reinforces the view that the effect of EE as a multidimensional cognitive enhancement strategy entails more than just the repeated exposure to novel sensory experience. Additionally, although there is evidence of visual attentional deficits in aged rodents (Harati et al., 2011; Jones et al., 1995; Muir et al., 1999; McGaughy and Sarter, 1995; Sarter and Turchi, 2002), our results during sample session suggest that the differences observed in the test cannot be attributed to perceptual or attentional deficits in the middle-aged animals but rather to an early object memory discrimination deficit in this group. This is consistent with work showing that implicit visual-perceptual memory appears to be unaffected by aging (Gabrieli, 1996).

Although both SOR and SLR task are known to rely on the medial temporal lobe, the SLR is proposed to be more heavily dependent on the hippocampus and the SOR on the perirhinal cortex (Miranda et al., 2017; Miranda et al., 2021; Aggleton et al., 2004). The lack of correlation between the improved SLR and SOR scores posterior to the intervention further suggest that these two tasks could rely on different neural substrates in middle-aged animals. The lack of neurogenesis in cortical regions key for object recognition memory points to a different EE dependent mechanism for s-SOR discrimination recovery than the one postulated for the HP-dependent spatial memory task. One possible mechanism for the object recognition memory recovery seen after EE exposure is the increase in cortical thickness, an effect of EE vastly reported in the literature (Diamond et al., 1972; Fares et al., 2013), and more recently shown in particular for the perirhinal/entorhinal cortex (Scholz et al., 2015). Additionally, previous reports indicate that cortical BDNF protein levels are correlated with exercise-induced improvements in non-spatial memory (Hopkins and Bucci, 2010). EE-induced long-term increase in task-induced BDNF levels could lead to the improvements shown in the similar version of the task.

We found changes in object exploration levels in the EE condition either on the sample or choice session of the tasks. Previous publications have described decreased spontaneous activity and reductions in anxiety-related behaviors due to EE (Chapillon et al., 1999; Fox et al., 2006; Wolfer et al., 2004) and attributed this to probable reductions in novelty seeking as a result of the abundance of sensory stimulations present in the EE (Brenes et al., 2009; Schrijver et al., 2002). However, the longitudinal nature of our EE experiment does not allow us to link this decrease to the continuous presence of objects inside the environmental enrichment context, since we cannot exclude the possibility of the decrease in exploration time being due to the prior exposure to objects as part of the protocol.

The importance of comparing the two streams of information capable of high cognitive stimulus discrimination (spatial and object domain) becomes critical given their translational value. Interestingly, recent studies in humans comparing both discrimination domains indicate that discrimination of similar object features is more sensitive to aging compared to similar spatial locations (Reagh et al., 2016) and that only the performance of memory for objects and not scenes, is predictive of early cognitive decline (Fidalgo et al., 2016). Studies found that aging particularly compromises the binding of different elements of an episode (Chalfonte and Johnson, 1996; Naveh-Benjamin, 2000; Old and Naveh-Benjamin, 2008). The present study provides key evidence to better understand the progression of BPS memory deficits in both spatial and object domain, as a part of early stages of the normal cognitive decline process. We evidence here that environmental factors contribute to shape BPS function in the object and spatial domain. Understanding how experience dependent plasticity (addressed here through the EE intervention) can contribute to a differential modulation of each of these cognitive domains could foster new theoretical developments and help in the design of non-invasive therapeutic strategies.

## Data Availability

The raw data supporting the conclusions of this article will be made available by the authors, without undue reservation.
