# Supplementary material for: Environmental enrichment in middle age rats improves spatial and object memory discrimination deficits
Source: Front Behav Neurosci. 2024 Oct 17;18:1478656. doi: 10.3389/fnbeh.2024.1478656 (PMC11528545; doi:10.3389/fnbeh.2024.1478656)
Supplement: Supplementary file 1 [file Data_Sheet_1.docx]

**Supplementary material**

**
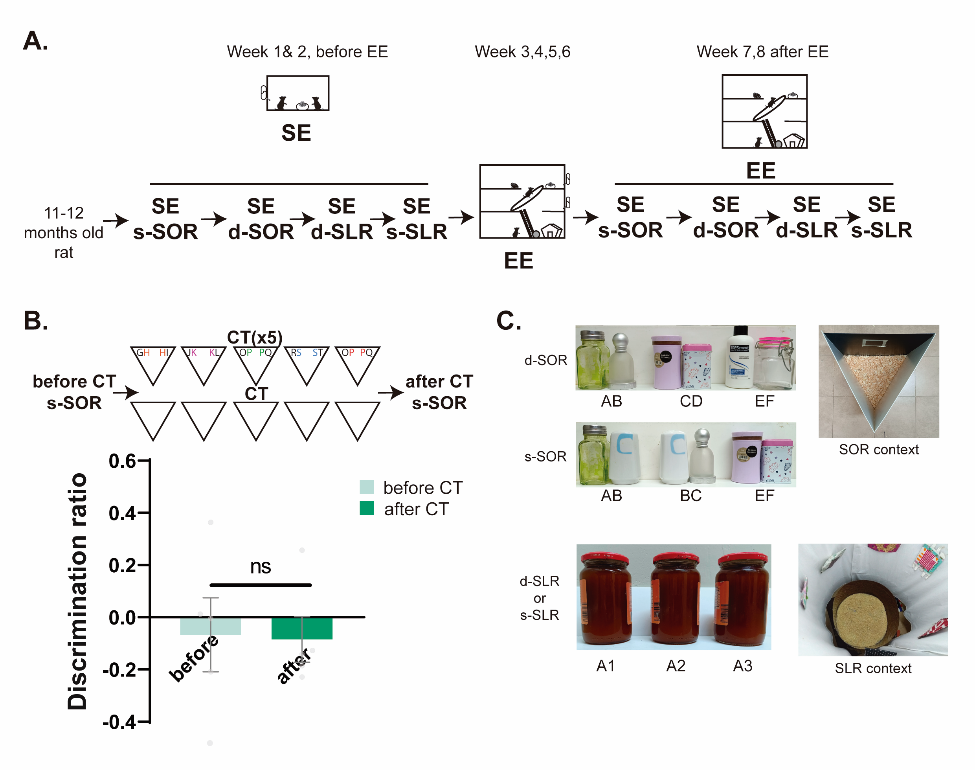
**

**Fig. S1. A.** Schematic protocol of the timeline of the EE experiment in Fig. 3. **B.** (Top) Schematic protocol of the timeline of the cognitive training (CT) experiment. Aged animals went through the similar version of the task (before CT) and went through a cognitive training protocol that consisted in 5 sessions of exposure to similar objects, after which they were retested again (after CT). (Bottom) Discrimination ratio of aged animals in the s-SOR task before or after CT. Paired t test t=0.124 p=0.907, n=5. **C**. (Left) Representative example of the combination of objects that would constitute a set of s-SOR and which are the conceptual differences with one that would constitute a d-SOR set. The two set of objects represented were never used for the same batch of animals since no object was shown twice to an animal. Instead, different sets of objects were used for each separate trial. The images are intended as a visual representation of the rationale behind the concept of “similarity” in terms of object identity. For the case of the SLR task, identical bulk objects were used and the “similarity” was not given by their identity but by the separation in their locations (smaller in s-SLR and larger in d-SLR). (Right) Images illustrating the enclosures used for the SOR task (up) and SLR task (down). Data expressed as the mean ± SEM; ns, non significant.

|  | **Ref** |  | **S-SOR** | **d-SOR** | **s-SLR** | **d-SLR** |
| --- | --- | --- | --- | --- | --- | --- |
| **Fig. 1** | **1.1** | **TR-Aged** | 104.00±.6.52 s | 94.34±6.36 s | 105.20±9.45 s | 122.70±10.03 s |
|  |  | **TS-Aged** | 56.08±3.58 s | 58.83+3.89 s | 42.73±5.12 s | 46.19±5.70 s |
| **Fig. 3** | **3.1** | **TR-SE** | 82.49±7.23 s | 78.49±3.56 s | 93.52±9.34 s | 94.19±7.15 s |
|  |  | **TR-EE** | 51.09±5.37 s | 56.31±7.46 s | 67.36±14.57 s | 62.67±14.90 s |
|  | **3.2** | **TS-SE** | 38.43±4.44 s | 37.45±2.20 s | 50.95±5.00 s | 36.37±5.91 s |
|  |  | **TS-EE** | 28.79±3.41 s | 31.62±5.27 s | 31.33 ±4.00 s | 24.45±3.42 s |

**Table S1.** Data represents total exploration time in seconds as Mean±SEM. TR, training session; TS, test session. Ref, reference in the main text.
